# Quantitative global studies of reactomes and metabolomes using a vectorial representation of reactions and chemical compounds

**DOI:** 10.1186/1752-0509-4-46

**Published:** 2010-04-20

**Authors:** Juan C Triviño, Florencio Pazos

**Affiliations:** 1 Computational Systems Biology Group, National Centre for Biotechnology (CNB-CSIC), C/Darwin, 3, Cantoblanco, 28049 Madrid, Spain; 2 Genomics Unit, National Centre for Biotechnology (CNB-CSIC), C/Darwin, 3, Cantoblanco, 28049 Madrid, Spain

## Abstract

**Background:**

Global studies of the protein repertories of organisms are providing important information on the characteristics of the protein space. Many of these studies entail classification of the protein repertory on the basis of structure and/or sequence similarities. The situation is different for metabolism. Because there is no good way of measuring similarities between chemical reactions, there is a barrier to the development of global classifications of "metabolic space" and subsequent studies comparable to those done for protein sequences and structures.

**Results:**

In this work, we propose a vectorial representation of chemical reactions, which allows them to be compared and classified. In this representation, chemical compounds, reactions and pathways may be represented in the same vectorial space. We show that the representation of chemical compounds reflects their physicochemical properties and can be used for predictive purposes. We use the vectorial representations of reactions to perform a global classification of the reactome of the model organism *E. coli*.

**Conclusions:**

We show that this unsupervised clustering results in groups of enzymes more coherent in biological terms than equivalent groupings obtained from the EC hierarchy. This hierarchical clustering produces an optimal set of 21 groups which we analyzed for their biological meaning.

## Background

The "genomic era" is being characterized by the massive determination of the molecular components of living systems. Genome sequencing projects are yielding complete genome sequences for hundreds of organisms [1]. Although not as massive as envisaged, structural genomics projects are speeding up the rate of protein structure determinations [2]. Many other initiatives also address large-scale repertories of molecular components and their relationships, seeking to decipher the "transcriptome" [3], the "interactome" [4,5], etc.

These massive data contain much information about living organisms. Studied as a whole, from a systemic perspective, they provide global pictures of different aspects of biology, which can help to answer very basic questions about how life evolved and how organisms do what they do with their "molecular toolkits". For example, the repertory of protein sequences (in the order of 10E7 known so far) and their evolutionary relationships (represented by amino acid sequence similarities) can be represented in a "sequence space" [6]. Studying this global landscape of protein sequences as a whole has produced important information on the estimated total number of protein families ("Nature's sequence toolkit"), functional groups, evolutionary relationships, etc. [7,8]. Similarly, the wealth of information on protein three-dimensional structures has allowed comprehensive hierarchical classifications such as SCOP [9] and CATH [10] to be generated. These classifications can subsequently be mined to perform global studies on the estimated total number of folds ("Nature's structural toolkit"), the evolution of protein structures, the relationship between sequence and functional spaces, etc. [11,12].

Metabolism was one of the last aspects to be studied from an "-omic" perspective [13]. The "metabolome" can be defined as the total complement of small-molecule metabolites within a cell, while the "reactome" is the set of chemical reactions that transforms these metabolites. In many cases, "metabolone" refers not only to the complement of small molecules but to quantitative measurements of their concentrations as well. An important difference from the "proteome" and other "-omics" is that most metabolic information has not been obtained by a high-throughput process but by the accumulation of knowledge over many years of detailed biochemical characterization of compounds, reactions and enzymes, knowledge that has been stored in databases such as KEGG [14] and BRENDA [15]. Only recently, high-througput techniques have been applied to the massive determination of metabolomes [16] and, even more recently, reactomes [17]. This has advantages and drawbacks. On the one hand, metabolic data are more reliable and do not suffer from the high degree of error common to high-throughput-omics data, such as the "interactome". On the other, biochemical knowledge can be biased to certain pathways and processes that have been more intensely studied than others.

One of the main problems in performing global studies of the reactome or the metabolome (aimed at characterizing "Nature's toolkit of biochemical capabilities"), equivalent to the studies discussed above for the proteome, is that there is no good way of representing and quantifying metabolic information. It is quite straightforward to quantify the similarity between the sequences or three-dimensional structures of two proteins. It is even possible to discretize these continuous scales of similarity to define intervals of evolutionary and functional relationship [18], expected structural similarity for a given sequence similarity [19], etc. The possibility of representing protein sequences and structures, as well as quantifying their corresponding similarities, is crucial for generating global classifications and subsequent global studies such as those cited above.

However, quantifying similarities among chemical compounds is not as straightforward as it is for protein sequences and structures. There are many different ways of quantifying similarities between small molecules [20], which can be used to study the global characteristics of the "metabolic space" [21] in a similar way to sequence and structure spaces. The main problem arises with chemical reactions (the "reactome"): there is no easy way of quantifying the "similarity between two (bio)chemical reactions". The few existing approaches [22-25] are not intended to generate detailed global classifications of biochemical reactions in an unsupervised way or to perform global studies of metabolomes and reactomes aimed at quantifying their chemical diversities. Indeed, the most widely-used classification of chemical reactions (the enzyme commission (EC) schema) is neither quantitative nor based on existing data. Rather, EC is based on an *a priori *imposed schema that organizes all existing enzymatic activities into a hierarchical classification of four levels with six main classes in the first level. It was designed mainly for nomenclature purposes and lacks a clear reflection at the molecular or evolutionary levels. Although very useful for many tasks, this classification was not designed for quantitative global classifications or comparisons of biochemical activities.

In this work, we propose a very simple way of representing chemical reactions in a vectorial form, which allows them to be compared quantitatively. This representation is based on an equivalent vectorial representation of chemical compounds, which imposes no *a priori *definition of functional groups or other "important" parts of molecules. It allows any chemical species to be represented in the same vectorial space. With this description, chemical compounds, reactions and pathways can be concomitantly represented in the same vectorial space. We demonstrate that the vectorial representation of chemical compounds, in spite of its simplicity, efficiently captures their molecular properties and can be used for predictive purposes. We used this method to generate an unsupervised global clustering of metabolic activities. This clustering makes more biological sense than equivalent groupings defined by the EC hierarchy, an improvement that is evaluated statistically using different criteria. Moreover, it results in an optimal set of 21 groups of reactions for which we look for their biological meaning.

## Methods

### Vectorial representation of chemical compounds

We looked for a metabolic representation that: (i) allows all chemical species to be represented in the same space regardless of their composition, molecular size, etc. (in order to compare and operate with them); (ii) imposes no *a priori *definition of important chemical features (functional groups, etc.), since a representation based on a pre-defined set of functional groups would necessarily restrict the results obtained to those that can be given in terms of those groups; and (iii) allows chemical reactions and pathways to be represented concomitantly in the same space (see next point).

**Figure 1:**
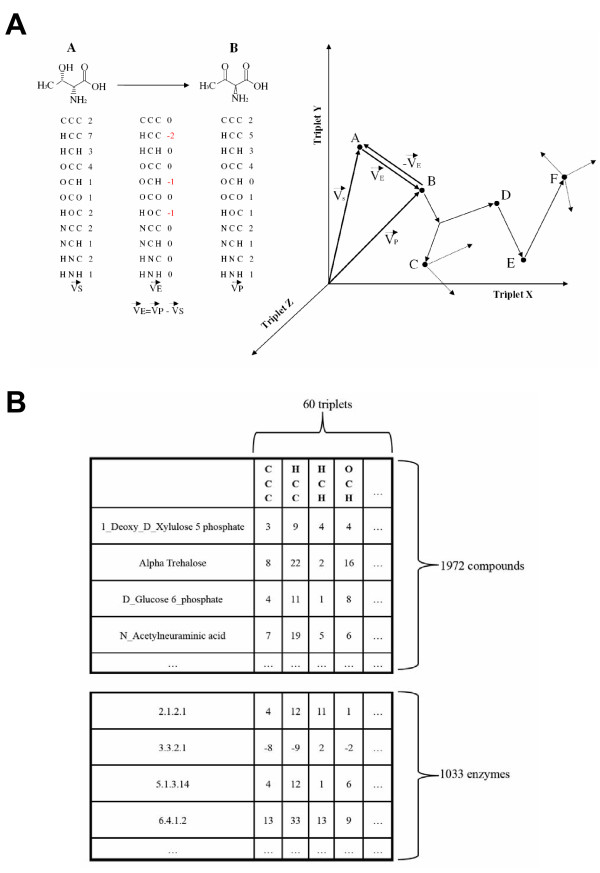
**Vectorial representation of chemical compounds and enzymatic activities**. (A) Example of an oxidation reaction (R-OH → R = O) included in KEGG. The components of the enzyme vector (which is the difference between the vectors of the product and substrate) to some extent reflect the nature of the transformation: loss of 2 HCC, 1 OCH and 1 HOC triplets. On the left, a 3D projection of the 60-dimensional space defined by the compound and enzyme vectors is depicted, with some compounds, reactions and pathways shown. (B) Representation of the vectors within the KEGG dataset. Both reactions and compounds are defined by 60-D vectors where each component represents an atom triplet.

We represent a chemical structure with a vector in which each component represents a triplet of neighboring atoms, and its value indicates the frequency of that triplet in the molecule (Figure 1). Using pairs of atoms instead of triplets would lead to shorter vectors (fewer possible combinations), but would not capture many aspects of the molecule's functionality; the chemical properties of an atom depend mostly on its neighbors up to distance 2. For example, the different properties of the carbonyl group C = O depend on the neighbors of the C: OH-CR = O (acid), H-CR = O (aldehyde), R-CR = O (ketone), etc. On the other hand, groups of 4, 5, ... neighboring atoms would produce vectors with too many components (many possible combinations). We previously used a similar representation to train a machine learning system for predicting the biodegradability features of chemical compounds [26] (see Conclusion).

This representation is not perfectly univocal, since in some cases different molecules can have the same vector (stereoisomers, positional isomers, etc.). Nevertheless, it provides a good balance between information content and utility for the goal of this work. The representation contains more structural information than is first apparent. For example the bond types, although not explicitly considered in the representation, are implicitly taken into account to some extent: as we go from hydrocarbons with few double and triple bonds (highly saturated) to hydrocarbons with many multiple bonds (unsaturated), we gain CCC triplets at the expense of HCH and HCC. In the "Results" section, we demonstrate that this representation captures important physico-chemical properties of compounds and can be used for predictive purposes.

### Vectorial representation of biochemical reactions

The vector for a reaction that transforms one chemical compound into another is just the difference between the vectors of the two compounds (product minus substrate) (Figure 1). The stoichiometry is taken into account by multiplying the compound vectors by the corresponding stoichiometric coefficients: e.g. for a reaction A -> 2B the reaction vector would be V_A->B _= 2·V_B _- V_A_.

Since this vectorial representation of reactions is based on that of the compounds, it inheres many of its characteristics: (i) any chemical reaction can be represented in the same space regardless of its characteristics (and hence operations and comparisons can be performed on them); (ii) there is no *a priori *definition of important reaction features. Moreover, the components of the reaction vector represent the triplets that are changed in the chemical reaction so they neatly encode the characteristics of the transformation (Figure 1). Another intuitive feature is that the vector of a given reaction is the opposite of the vector of the inverse reaction (V_A->B _= -V_B->A_) (Figure 1).

In this representation, all chemical reactions have to be simplified as transformations of one single compound into another. For a reaction with more than one reactant and/or product, a "main" pair substrate ->product has to be chosen and information about the other substrates/products becomes part of the vector components. This is not a problem since in metabolism we can usually define a "main transformation" and as such is annotated in metabolic databases (see "Datasets" below).

So, in this multidimensional "metabolic space", chemical compounds are points (or the corresponding vectors), chemical reactions can be seen as arrows (difference vectors) going from one point (substrate) to another (product), and biochemical pathways can be seen as sets of consecutive reaction vectors (a "walk" within this space) (Figure 1).

### Datasets

We took from KEGG [14] all the metabolic reactions annotated for the model organism *E. coli*. For each reaction, the substrate→product transformations annotated as "main" in the "RPAIR" field in KEGG are taken. The molecular structures for the two compounds involved in this transformation are also retrieved from KEGG in .mol format. Hydrogens are added with the OpenBabel package http://www.openbabel.org. Open Babel is also used to transform the .mol files into .pdb since the bond information required to calculate the triplets is easier to obtain from .pdb files. Finally, we calculate the two triplet vectors for the compounds, and the corresponding triplet vector for the reaction as the difference (Figure 1). We end up with 1972 compound vectors and 1033 reaction vectors. We can have more than one vector for a given EC code, depending on whether that enzyme activity is associated with (acting in) more than one reaction.

Both reaction and compound vectors are defined by 60 components (triplets). These triplets were not imposed *a priori*. They are the ones with the highest frequencies in our dataset. We excluded some triplets with very low frequency, involving rare atoms and appearing in very few compounds. Some triplets involve "unknown" atoms ("X"). These come from molecules in KEGG for which some parts are not detailed at the atomic level but indicated by a general group "-R". These "R" groups (and hence our "X") usually represent biological polymers (peptides, poly-sugars, DNA, etc).

In order to study the relationships between our vectorial representation of reactions and their functional characteristics, additional information on the enzymes catalyzing these reactions was obtained from other resources. From BRENDA [15] we retrieved the "reaction type", a set of one or more keywords describing the transformation carried out by the enzyme. The total number of unique keywords in this dataset is 165. From BRENDA we also retrieved the PROSITE motifs associated with the enzymes. PROSITE [27] is a database of short sequence motifs. We retrieved 183 unique PROSITE motifs associated with the enzymes in our dataset. Interpro [28] is a resource that integrates information from diverse databases defining protein domains according to different structural and sequence criteria, such as Pfam, Prodom, SMART, SCOP, etc. From Interpro, we retrieved 1107 unique signatures (domains) associated with the enzymes in our dataset. Gene Ontology [29] defines three sets of terms for representing three different and independent aspects of the complex phenomena of protein function ("molecular function", "biological process", and "cellular compartment"). These terms are related by parenthood relationships defining three hierarchies which go from very general to more specific terms. A protein (gene product) becomes annotated by associating it to one or more of these terms. From Gene Ontology we retrieved the GO terms in the "molecular function" (GO:MF) and "biological process" (GO:BP) sub-ontologies for our enzymes: 736 GO terms (439 GO:MF and 297 GO:BP). In the following, we use the general term "keyword" referring to BRENDA keywords, PROSITE motifs, Interpro domains and GO terms.

Additionally, to study in more detail the relationships between the compound vectors and the physico-chemical properties of the molecules they represent, a supplementary set of chemical compounds with associated physico-chemical information was obtained from http://cheminformatics.org/ That dataset was previously used by Karthikeyan et al. to develop a melting-point prediction method [30]. The molecules in it are provided in SMILES format http://www.daylight.com/smiles. We convert from SMILES to .pdb with OpenBabel, and then to triplet vectors as explained above. For this dataset, owing to the more diverse atomic compositions of the molecules, we needed 96 atom triplets. From the original 4450 molecules in the dataset, 43 could not be represented in vectorial form for several reasons (e.g. SMILES not readable by OpenBabel), resulting in a final dataset of 4407 compounds. Hence, our final dataset contains 4407 molecules with very diverse chemical properties; e.g. the range of molecular weights is 84-815 and the range of logP is -3.18 to +14.16. We used three physicochemical properties in this work: logP (log ratio of concentrations between octanol and water, a measure of hydrophobicity); MW (molecular weight); and TPSA (Topological Polar Surface Area, the surface of all polar atoms). The program SPSS was used to perform a multivariate linear regression analysis to relate the compound vectors to these chemical properties. The WEKA package [31] was used to perform classification studies aimed at evaluating the predictive value of this vectorial representation. For that, the dataset of 4407 compounds was randomly divided into 10 subsets. Nine of these (training set) were used to perform the linear regression analysis, and the result was then used to predict the chemical property of the remaining group (test set). Cycling this procedure for the other nine groups allowed predictions for all the compounds to be obtained. The predicted values for the three properties were compared with the experimental values by linear correlation.

### Clusters of reaction vectors

A distance tree for all the reaction vectors was generated by the UPGMA algorithm implemented in the R package http://www.r-project.org, based on the Euclidean distances between the vectors. Cutting this tree at different levels (going from the root to the leaves) produces different groupings with increasing number of clusters. The "Mclust" function of the "mclust" library of the same package was used to calculate the optimal number of clusters. This function is based on the BIC parameter ("Bayesian information criterion"). We explored from 1 to 100 clusters and the optimal number according to BIC criteria was 21.

We compared this clustering with equivalent classifications defined by the EC hierarchy using the enzyme annotations in the BRENDA, PROSITE, InterPro and GO resources (See "Datasets" above). For our reaction vectors (reactome) we can have any number of clusters depending on the level at which we cut the UPGMA tree, although the optimal number of clusters is 21. On the contrary, the EC classification defines four possible groupings only, depending on the EC level (6 clusters at the 1^st ^level, 39 at the second, etc.). In order to compare EC with reactome for equivalent number of clusters we obtain the groupings of the reactome with the same number of clusters (6, 39, ...).

For a given set of keywords (BRENDA, GO, Interpro, ...) and a given clustering we created a contingency table containing the frequencies of each "keyword" (annotation) in each cluster, that is, the number of enzymes within that cluster associated with the keyword. A chi-squared test was applied to each contingency table. We used the Yates correction [32], which is applied to account for the low frequencies of some keywords and results in a more conservative test. Since different classifications have different degrees of freedom, we convert the chi-squared values (Χ^2^_i_) to z-scores (z_i_) using Fisher's approximation [33], which approximates the chi-squared distribution by a normal distribution with mean 0 and σ = 1.

where *n*_i _are the degrees of freedom: (N-1)*(k-1), k being the number of keywords in each dataset and *N *the number of clusters. A high z-score indicates a relationship between the clustering and the set of keywords: elements (enzymes) clustering together tend to have the same keywords, and vice versa.

To elucidate the biological meanings and characteristics of the 21 groups which form the "optimal" clustering of the reactome (see above), we extracted the keywords and the components of the reaction vectors (atom triplets) specifically associated with each. We did that by calculating, for each keyword/triplet in each of the 21 clusters, the p-value of its frequency within that cluster, taking into account the background frequency of that keyword/triplet. For that we used the hypergeometric distribution and the Poisson distribution for low frequency cases (n*p < 5 and *p *< 0.1, *n *being the frequency of the keyword/triplet in the cluster and *p *the probability of that keyword/triplet in the 21 clusters) [34].

## Results

### Assessment of the compound vectors

As observed in the Discussion, there is a plethora of methods for representing chemical compounds in ways manageable by computers. It is not the objective of this work to propose another description and compare it with existing ones. Nevertheless, we obviously have to evaluate the ability of this description to capture the chemical properties of compounds. We do that by multivariate linear regression analysis using the chemical property as dependent variable and the components of the vector (atom triplets) as independent variables. We apply this analysis to a database of 4407 chemical compounds and to three different chemical properties: hydrophobicity (logP), molecular weight (MW) and polar surface area (TPSA). See Methods for a full description of the procedure.

Table 1 contains the R and R^2 ^parameters of the linear regression for the different properties. These values clearly show that these properties are well characterized in the vectorial representation used.

**Table 1:** Parameters of the multivariate linear regression between three chemical properties and the components of the compounds vectors.

	R	R^2^
**logP**	0.902	0.814

**MW**	0.977	0.955

**TPSA**	0.977	0.954

R is the Pearson correlation coefficient.

**Figure 2:**
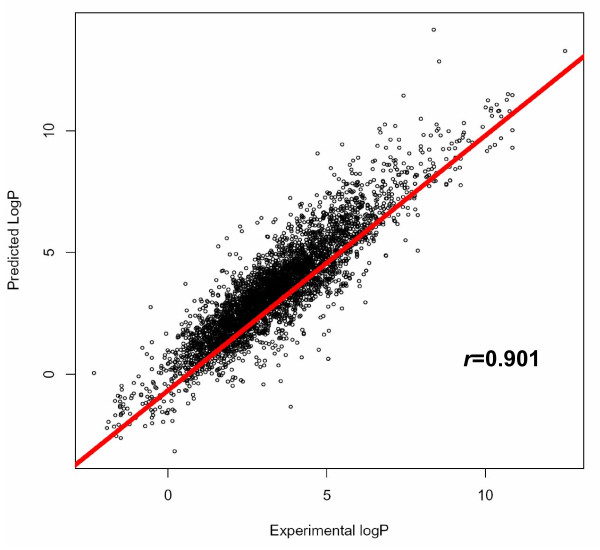
**Correlation between experimental and predicted hydrophobicity**. Correlation between the experimental and predicted hydrophobicity (quantified by the logP value) for the dataset of 4407 molecules.

Such a clear relationship between the compound vectors and the chemical properties can be used for predictive purposes. As described in Methods, we re-calculate the linear regressions using 9/10 of the vectors and use that regression to predict the chemical properties of the remaining 1/10, cycling these sets in a 10-fold cross-validation. Figure 2 shows the relationship between the predicted and experimental logP for the chemical compounds. The predicted logP values tend to be similar to the experimental ones (*R *= 0.901). The results of the predictions for the other two properties are available as additional material (additional file 1). These results are better than the ones for logP, as shown by the correlation (*R*) values (0.977 for TPSA and 0.977 for MW). Nevertheless, in spite of their methodological value, these results are not useful for a real-world application (it makes no sense to "predict" the molecular weight of a chemical compound). That is why we focus on the results for logP here in spite of being slightly worse, since these are the more interesting ones.

### Hierarchical clustering of the reaction vectors for the *E. coli *metabolome ("reactome")

**Figure 3:**
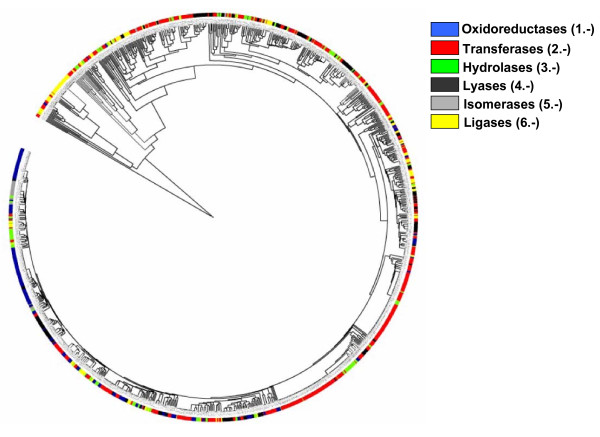
**Hierarchical clustering of the reaction vectors**. The tree is constructed using the UPGMA method based on the Euclidean distances between the reaction vectors. The main EC class of the associated enzymes is represented by a color code. The representation was generated with iTOL [38].

Figure 3 shows the UPGMA tree based on Euclidean distances defined by the 1033 reaction vectors in the metabolome of *E. coli*. The color code represents the first digit of the EC code of the corresponding enzymes.

It can be seen that, in general, there is agreement between the EC and the vector-based classifications; clusters of similar vectors tend to contain enzymes with the same EC 1^st ^number. Nevertheless, vectors with the same EC number can be far apart in this classification. There are other important discrepancies, highlighting the value of the method presented here for quantifying similarities between enzymes. For example, there is a group of reactions with identical vectors (distance = 0) carried out by very different enzymatic activities according to the EC classification: 3.5.1.19, 3.5.1.1, 6.3.5.4, 6.3.5.5, 6.3.1.2, 2.4.2.14, 3.5.1.2 and 2.6.1.16. These enzymes, spanning three of the six main EC classes (hydrolases (3.-), ligases (6.-) and transferases (2.-)), are all nevertheless involved in the same chemical transformation, the conversion of an amide to a carboxylic acid: R-CONH_2_→R-COOH. So our vectorial representation captures a functional similarity between these enzymes that is not represented by the EC classification. Another interesting example is the group representing enzymes involved in phosphate hydrolysis. These belong to three main EC classes (2.-, 3.- and 4.-) but are clustered together in our tree. The hydrolysis of a pyrophosphate group is represented by another cluster, distant from the first, reflecting the different natures of these two processes.

This enzyme tree, in newick format, is available as supplementary material (additional files 2 and 3).

### Assessment of the biological significance of the clustering in comparison with the EC classification

We compared this clustering with the one defined by the EC classification for different number of clusters and according with different descriptors of biological significance (see Methods). Table 2 shows the z-scores of the Fisher test for different clustering schemas and different sets of keywords. A z-score close to 0 (or lower than the standard threshold of 2.0) indicates no relationship between the clustering and the set of keywords, these keywords being uniformly distributed across all clusters. It can be seen that both clustering schemas have very high z-scores for all sets of keywords, meaning that both schemas group similar enzymes together (according to the "similarity" criteria implicit in these sets of keywords). Nevertheless, the z-scores for the reactome clusters automatically obtained from our vectorial representation are much higher than the equivalent ones defined by EC, and this is the case for all sets of keywords. This means that the reactome clustering is more meaningful in biological terms. The dependence between clusters and keywords is higher for this reactome clustering, which is especially striking because some of these sets of keywords, defined by experts, are partly based on the EC classification. This is the case for BRENDA keywords, and is reflected in the observation that EC has higher z-scores (and lower differences with reactome) with BRENDA than with any of the other three datasets (Table 2). Perhaps BRENDA shows the lowest difference between the two clustering schemas, and is least in agreement with the reactome clustering (Table 2), because its keywords are partially based on EC.

**Table 2:** Fisher's z-scores representing the dependence between a given clustering and a set of keywords.

EC level	GO			Interpro			BRENDA			Prosite		
	**#clusters**	**reactome**	**EC**	**#clusters**	**reactome**	**EC**	**#clusters**	**reactome**	**EC**	**#clusters**	**reactome**	**EC**
	
1st (X.-.-.-)	6	223.10	48.62	6	322.88	42.53	6	118.29	65.73	6	178.76	61.90
2nd (X.X.-.-)	36	1458.93	611.98	39	2163.93	905.54	39	334.23	329.99	33	455.45	316.99
3rd (X.X.X.-)	79	2325.21	1660.40	79	3438.31	1948.35	108	1236.48	1017.59	67	783.31	600.11

**-**	**21**	**299.97**	**-**	**21**	**468.07**	**-**	**21**	**93.77**	**-**	**21**	**276.52**	**-**

The optimal reactome clustering with 21 groups, for which no equivalent exists in EC, is also included. Some EC groupings results in clusters without any enzyme associated to keywords in that particular dataset. These are excluded from the analysis and are responsible for the small variations in the number of clusters (e.g. 33, 36, 39 for the 2^nd ^level). For comparison, the reactome is clustered with the same (reduced) number of clusters.

### Characterization of the 21 clusters

According to the BIC criteria (see Methods), the optimal number of enzyme clusters within our dataset was 21. We investigated the characteristics of these groups. We did that by looking for keywords (within the four different datasets) and components of the enzyme vectors (atom triplets) that are differentially associated with each of the 21 clusters. For each keyword and each triplet we calculated a p-value that quantifies the statistical significance of their association with a given cluster (see Methods). The table with detailed information on the significant keywords/triplets for the 21 clusters is available as Supplementary Material (additional file 4). The biological meanings of most of the clusters can be elucidated on the basis of their associated keywords/triplets. For example, cluster #1 is related to processes involving transfer/elimination of relatively large groups, as indicated by triplets involving C-C and C-O bonds (CCC, OCC, OCO) and keywords such as "elimination" and "condensation" (BRENDA), "*transferase", "*synthase" (GO, Interpro and Prosite). Cluster #2 seems to be related to the addition of amino acid groups. Cluster #3 is clearly related to redox reactions: triplets involving C-O, C-H and O-H bonds, "oxidation", "redox reaction" (BRENDA), "oxidoreductase", "NAD binding", "iron/sulphur cluster" (GO), etc. Cluster #6 is related to nucleoside formation via N-glycosyl bonds. Cluster #4 seems to be related to sulfur metabolism. Cluster #7 is related to acyl group transfers and CoA ligation. Cluster #10 is related to UDP-N-acetylmuramate metabolism (peptidoglycan and cell wall formation), so it could be specific to bacterial metabolism. Clusters #16 to #19 are all related to different aspects of phosphate group transfer, reflecting the importance of this process for the cell. But each cluster seems to reflect a different aspect of this general process: while cluster #18 seems to be related to the transfer of pyrophosphate (diphosphate) groups, cluster #19 is related to hydrolysis of phosphate groups and #16 and #17 to metabolic kinases (#16 being related to addition of phosphate and #17 to removal). Clusters #5, #20 and #21 could be methodological "artifacts" arising from the way molecules are represented in KEGG and the way we translate them into atom triplets. In KEGG, large groups are sometimes represented by "R-", without specifying their constituent atoms. These are considered single atoms of unknown character by our system and represented by "X" (see Methods). So in our vectors we have some triplets involving "X". The three clusters mentioned above are associated with these triplets. Nevertheless, since most of the "X" represent large transferable groups ("R-" = peptides, sugar polymers, DNA fragments, ...) these clusters tend to reflect processes involving transfers of large polymers, so they retain some biological meaning.

Thus, these groups of reactions/enzymes detected in the "reactome" in an unsupervised manner provide a global view of the main processes in *E. coli *metabolism.

## Discussion

There are two main approaches to constructing biological classifications, which could be termed "top-down" and "bottom-up", or "supervised/unsupervised" [35]. Top-down approaches define a set of classes *a priori*, usually based on expert knowledge, and then assign the objects to them. In contrast, bottom-up approaches construct the classification ("clustering" in this case) from the data in an unsupervised way, i.e. hierarchically clustering the data and looking for the resulting groups. Both approaches have advantages and drawbacks. For protein sequences and structures we can find examples of both approaches, e.g. SCOP [9] (supervised) vs. Dali/FSSP [36] (unsupervised) for classifying protein 3D structures.

For metabolic data, the top-down (supervised) approach dominates. The main schema for classifying biochemical reactions (EC) is very valuable for nomenclature purposes, but in many cases it is not clearly reflected at the molecular level. While other ways of classifying chemical reactions based more on molecular details (i.e. reaction mechanisms) are being developed [37], the EC classification is widely extrapolated beyond its primary goal (nomenclature) on the assumption that it fully reflects molecular details. The main problem for constructing bottom-up classifications of metabolic phenomena may be that there is no standard way of quantifying similarities among chemical compounds and, more especially, metabolic reactions, in contrast to protein sequences and structures.

There are many methods for representing the structures of small molecules and quantifying their similarities, each intended to a particular purpose [20]. Actually this lack of standard shows that the issue more complex than in the case of proteins. Some of these representations have been used to construct bottom-up classifications of the "metabolome" [21], but this pioneering and interesting work has some drawbacks. For example, it represents metabolic compounds by "imposing" the functional groups considered important. From the outset, this restricts the kind of results that can be obtained. The system classifies metabolites on the basis of the functional groups we *a priori *decide are important. Such "supervision" might hinder the discovery of features that are not related to those functional groups.

The situation is worse for the "reactome". There are few approaches to quantifying the similarities among biochemical reactions. These approaches either tackle very specific aspects of chemical transformations and have been applied to small sets of reactions [22,25], or do not allow chemical compounds to be represented in the same space so that relationships between the "reactome" and the "metabolome" can be studied [23], or are aimed at measuring similarities in mechanism rather than the transformation itself [24]. For these reasons, although these approaches produced very interesting results for specific tasks, they are not easily applicable to the task of generating general global classifications of reactions.

## Conclusion

As far as we know the work presented here is the first that aims to classify (cluster) the "reactome" in an unsupervised (bottom-up) way without any imposition of the kind of transformation or functional groups involved.

It is not the objective of this work to propose another way of representing molecular structures and compare it with existing ones. The main goal is to allow chemical compounds, enzymatic activities and pathways to be represented concomitantly in the same vectorial space. Since we wish to unravel the global properties of the reactome, we want to keep our representation as simple as possible (i.e. not based on functional groups) so that the kind of results we can obtain is not restricted. We show that, in spite of its simplicity, this representation captures important physicochemical properties of the molecules. The representation of chemical compounds we use here does not impose any definition of "important groups". We have previously used a similar representation for predicting the environmental fate of chemical compounds [26]. That representation included information on the bond type in the triplets, as well as the molecular weight and the water solubility of the compounds. These "heterogeneous" vectors with components representing different things and in different scales (triplet counts, solubility, MW), are not the most adequate for representing the chemical reactions in the same space. Moreover, our results show that the information on the water solubility and the MW is implicit in the atom triplets.

The vectorial representation of chemical reactions used in this work does not take into account some characteristics of chemical transformations. For example, it disregards stereochemistry, and aspect known to be very important in some catalyzed reactions. On the other hand, it is general enough to represent and study whole reactomes.

We show that the groups of enzymatic activities arising from the unsupervised clustering of the *E. coli *reactome are more significant than the equivalent EC clusters and have biological meaning. We can think of these groups as representing the main trends of the metabolic landscape of this organism. The presence of many groups related to phosphate metabolism is interesting, highlighting the importance of this process for the cell. The presence of a group devoted to reactions related to peptidoglycan metabolism shows that these results are organism-dependent, and opens interesting possibilities for comparing reactomes between different organisms.

We think that this new representation of chemical reactions opens many possibilities for the global study of Nature's metabolic capabilities. In the same way that global classification of the spaces of protein sequences and structures provided important biological information, studies of metabolism based on global classifications such as the one proposed here may also provide valuable data.

## Authors' contributions

FP conceived the original idea. JCT implemented the idea and carried out the tests. FP and JCT wrote the paper. All authors read and approved the final manuscript.

## Supplementary Material

Additional file 1 Correlation between experimental and predicted molecular weight (MW) and total polar surface area (TPSA). Click here for file

Additional file 2 **UPGMA tree of reaction vectors, in newick format.**  This file can be viewed with most phylogenetic tree viewers, including the on-line tool iTol (http://itol.embl.de). Click here for file

Additional file 3 File with the information needed for coloring the tree "Add2_UPGMA.nw" in iTOL (itol.embl.de) according to EC number. Click here for file

Additional file 4 Keywords associated with the 21 clusters. Click here for file
