# Supplementary figures and images for: Quantitative global studies of reactomes and metabolomes using a vectorial representation of reactions and chemical compounds
Source: BMC Syst Biol. 2010 Apr 20;4:46. doi: 10.1186/1752-0509-4-46 (PMC2883543; doi:10.1186/1752-0509-4-46)

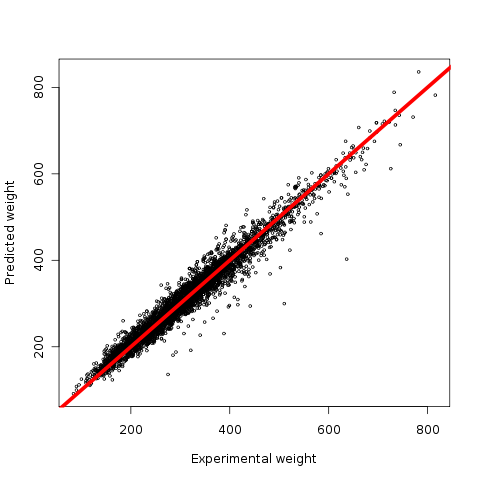


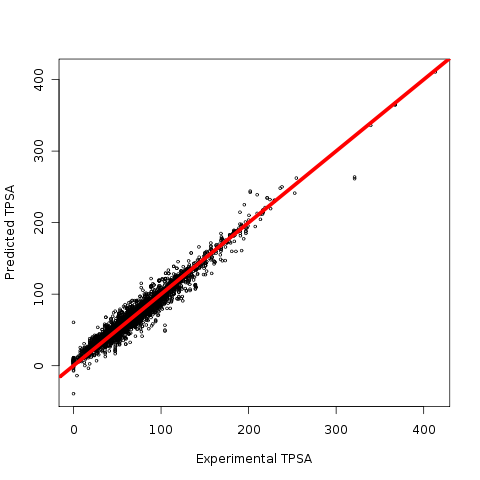

Supplement: Additional file 1 — Correlation between experimental and predicted molecular weight (MW) and total polar surface area (TPSA). [file 1752-0509-4-46-S1.DOC]
